# Ecological factors associated with fox feces density in an *Echinococcus multilocularis* endemic zone in Japan

**DOI:** 10.3389/fvets.2024.1387352

**Published:** 2024-11-05

**Authors:** Megumi Fukui, Kohji Uraguchi, Himika Numa, Toru Suzuki, Michiko Karasawa, Kaoruko Maita, Terumi Yokozawa, Yoko Hayama, Kohei Makita

**Affiliations:** ^1^Veterinary Epidemiology Unit, Department of Veterinary Medicine, School of Veterinary Medicine, Rakuno Gakuen University, Ebetsu, Hokkaido, Japan; ^2^Hokkaido Institute of Public Health, Sapporo, Hokkaido, Japan; ^3^Department of Environmental and Symbiotic Sciences, College of Agriculture, Food and Environmental Sciences, Rakuno Gakuen University, Ebetsu, Hokkaido, Japan; ^4^Division of Transboundary Animal Disease Research, National Institute of Animal Health, National Agriculture and Food Research Organization, Tsukuba, Ibaraki, Japan

**Keywords:** echinococcosis, feces count, red fox, INLA, spatio-temporal autocorrelation

## Abstract

**Introduction:**

Human alveolar echinococcosis caused by *Echinococcus multilocularis* is an important zoonotic disease in the northern hemisphere. The life cycle of *E. multilocularis* is maintained primarily in wild animals and requires an intermediate host (mainly small mammals). Human can become an intermediate host through accidental ingestion of *E. multilocularis* eggs. Hokkaido Prefecture is the only area of Japan in which human alveolar echinococcosis is endemic. The purposes of this study were to elucidate the land use ecological factors associated with the density of red fox feces along paved roads and the relationship between the distributions of red fox (*Vulpes vulpes*) populations and fox feces, which determine the level of hazard from eggs.

**Methods:**

A series of surveys was conducted in the central part of the Nemuro Peninsula of Hokkaido, excluding urban areas, over a total of 4 years in May–June in 2014 and 2016–2018 when red foxes remain with their cubs around the dens. Transects of 500 m were set up on paved roads, and feces within the transects were counted. Univariable and multivariable analyses were performed to examine ecological factors including the principal components (PCs) of five land use–type occupancy proportions within 500 m and 1 km, respectively, as explanatory fixed-effect variables. The number of feces in each transect was examined as the response variable using integrated nested Laplace approximation with negative binomial errors with a spatio-temporal autocorrelations structure to separate the effects of similarities of neighboring locations and annual variation. The multivariable models with the lowest widely applicable information criterion values were selected.

**Results:**

The feces density was explained by the PC of the 500- m buffer (−0.27, 2.5th and 97.5th percentiles: −0.44, −0.10) characterized by mixed forests (−0.82) and scarcity of residential areas (0.29) and the proximity to the nearest livestock farm house (−0.35, 2.5th and 97.5th percentiles: −0.53, −0.17). This suggested that foxes defecate in the areas where prey is abundant, avoiding humans.

**Discussion:**

Policy discussions regarding bait distribution design targeting these conditions should be initiated.

## 1 Introduction

Human alveolar echinococcosis caused by *Echinococcus multilocularis* is an important zoonotic disease in the northern hemisphere and a neglected zoonotic disease according to the World Health Organization (1). Genus *Echinococcus* taxonomically belongs to phylum Platyhelminthes, class Cestoda, order Cyclophyllidea, family Taenidae (2). *Echinococcus* had a long history of taxonomic and nomenclatural confusion, but recent application of molecular tools in addition to morphological and ecological criteria brought widespread agreement that *Echinococcus* should be split into 10 species. According to the criteria, *E. multilocularis* is the only species which causes alveolar echinococcosis in humans (3). The life cycle of *E. multilocularis* involves foxes as the definitive host and to a lesser extent dogs, cats, coyotes, and wolves and their rodent prey (intermediate hosts) in ecosystems generally separate from humans (4).

**Figure 1:**
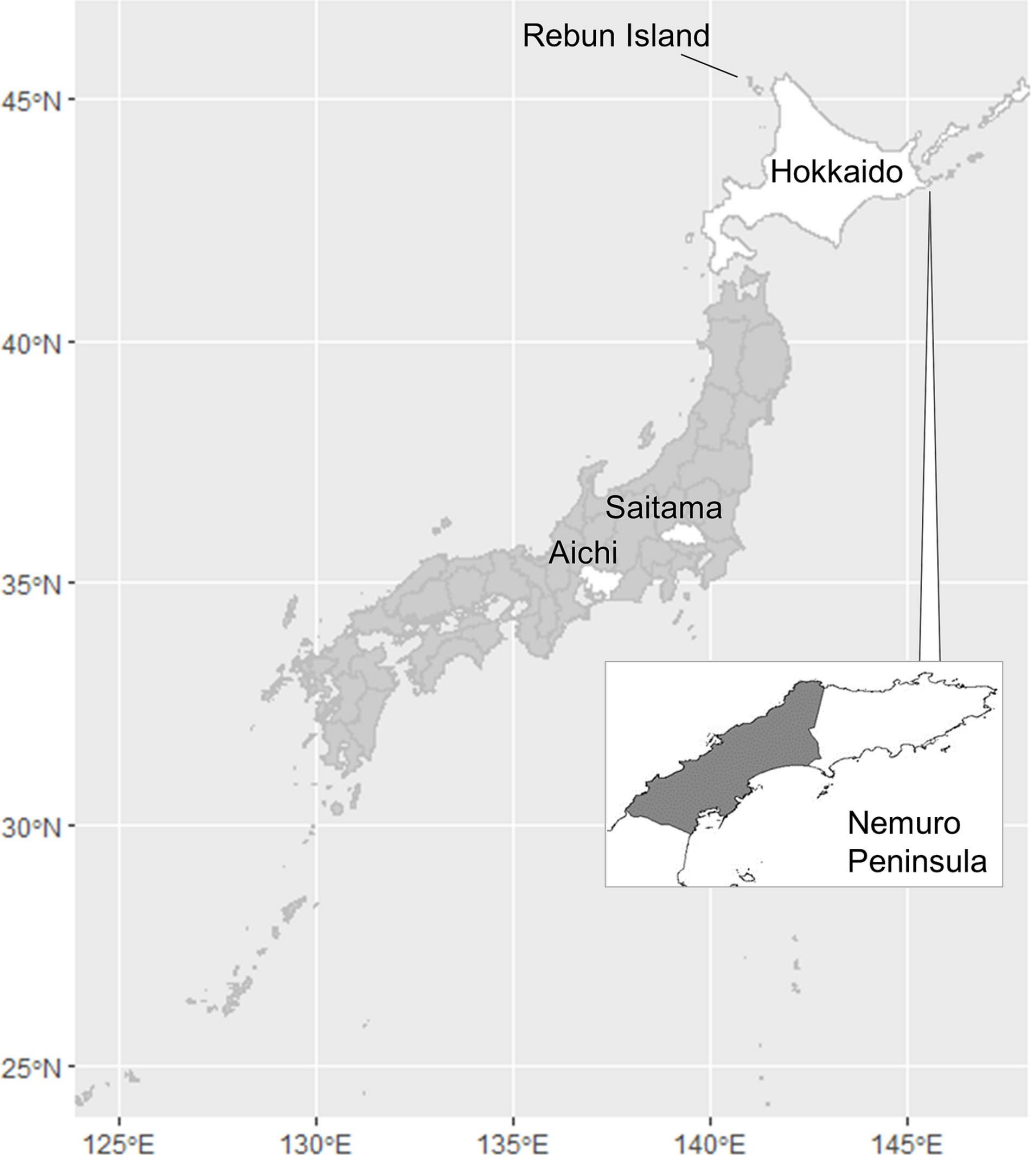
Map of Japan indicating the locations of Aichi, Hokkaido, and Saitama Prefectures, the Nemuro Peninsula, and Rebun Island. The shaded portion of the Nemuro Peninsula represents the study area.

Hokkaido Prefecture is the only area of Japan in which human alveolar echinococcosis is endemic (Figure 1). In this prefecture, the life cycle of *E. multilocularis* is maintained between the final host, the red fox (*Vulpes vulpes schrencki*), and the intermediate host, voles (*Myodes rufocanus bedfordiae*) (5). The definitive host ingests larvae through predation of intermediate hosts that became infected via ingestion of eggs excreted by the final host. In the intermediate host, larval growth continues indefinitely in the liver in the proliferative stage (4). Humans, as aberrant intermediate hosts, become infected following the accidental ingestion of *E. multilocularis* eggs (6). After a period of slow larval growth, the numerous tiny cysts within the liver can cause a lethal pathophysiology similar to liver carcinoma. Unfortunately, early diagnosis of alveolar echinococcosis is very difficult because of the long latent or asymptomatic period, which can span as long as 20 years (7). The only effective treatment for alveolar echinococcosis is surgical resection, and > 90% of patients die if the disease is left untreated (8). Moreover, helminths are known to regulate host immunity (9) and to hinder any vaccines from providing optimal protection (10). The disease burden in humans may be therefore greater than that directly observed in the case counts.

**Figure 2:**
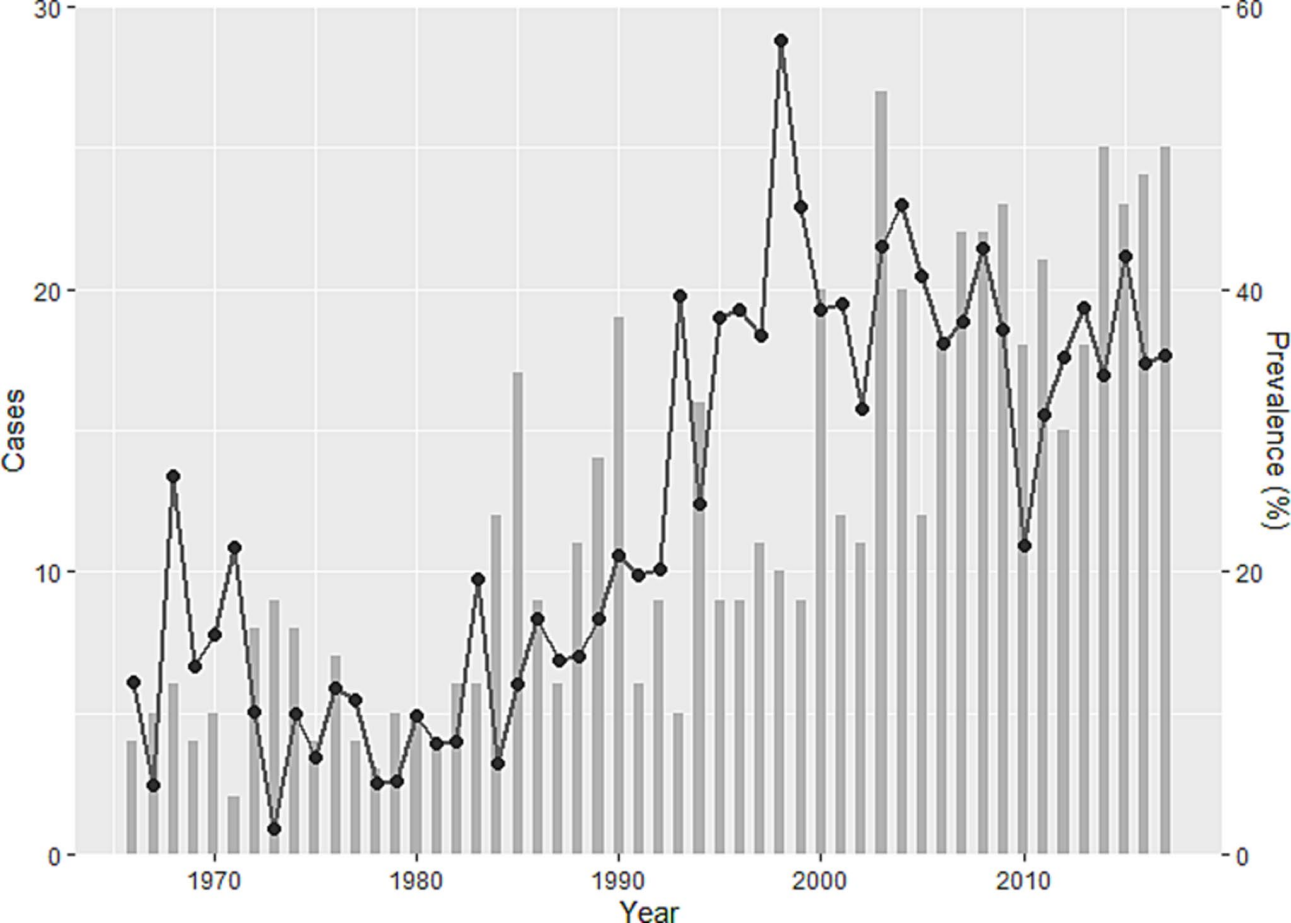
Trends in human alveolar echinococcosis cases (bar chart) and prevalence of *Echinococcus multilocularis* in foxes (line) in Hokkaido Prefecture between 1966 and 2017 (15)). The bar chart shows cases, and the line shows the temporal change of prevalence.

Incursion of human alveolar echinococcosis into Hokkaido Prefecture has been reported twice. The first incursion occurred in 1937 on Rebun Island (Figure 1) (11). The disease was successfully eliminated in the island by 1970. The second incursion was reported in the Nemuro Peninsula, the study site in this paper, in 1965 (12). Human cases of the disease were restricted within this region until 1979, but thereafter, the geographic distribution of cases expanded to cover all of Hokkaido (13). This geographic expansion of the disease in humans was accompanied by a gradual increase in the prevalence in red foxes, reaching 57.4% in 1998 (Figure 2) before stabilizing at approximately 40% in the early 2000s according to the monitoring of Hokkaido Prefectural Government (8, 14). Human cases also increased and since 2000, approximately 20 new human cases have been reported annually in Hokkaido Prefecture (15). The age of the infected individuals has ranged between 7 and 81 years, with the highest frequency in those 40–60 years old (16). Important risk factors in Hokkaido Prefecture include livestock farming (cattle and pigs) and the use of well water (17). Moreover, dairy farmers, fishermen, civil engineering workers, and individuals involved in outdoor jobs are at higher risk of infection (6). In recent years, cases of canines infected with *E. multilocularis* have been reported in Saitama (18) and Aichi Prefectures (19) on Honshu, the main island of Japan (Figure 1). The geographical expansion of *E. multilocularis* has been observed in Europe in different contexts. In addition to foxes, wild invaders such as racoon dog play increasingly important roles in transmission (20, 21). The urbanization of *E. multilocularis* has emerged in Europe, and fox density can be larger in urban than in rural areas, suggesting enhanced chance of contact between humans and infected fox feces (21, 22). In Hokkaido, Japan, the increase of urban foxes has been recognized (23), and racoon dogs are already known to be infected with *E. multilocularis* (24).

For zoonotic helminths including echinococcosis, establishing diagnostic capability, understanding epidemiology including transmission and wildlife habitat dynamics, border security and surveillance, understanding culture are important (21). The key strategy for reduction of prevalence in red foxes is currently anthelmintic bait distribution (23). Vaccine development against helminths is challenging, primary due to economic reason (21), but also the accessibility of the tissues in which the helminths reside (9). However, new technologies such as organoids and single-cell sequencing may facilitate development of helminth vaccines (9). Even though such new tools may become available, the effective control of echinococcosis must take One Health approach (21, 25, 26).

Regarding the disease control, the Hokkaido Prefectural Government established the Hokkaido Prefecture Echinococcosis Control Council, which provides services such as hygiene education and medical examinations, development and dissemination of early detection techniques (serum diagnosis), improvements to the water supply, and implementation of measures to control host animals. With regard to measures to control host animals, the Hokkaido Institute of Public Health supervises the distribution of anthelmintic-containing bait for foxes and has conducted effectiveness tests of baits prepared by the local municipalities since 1999 (27, 28). In the effective tests conducted in Nemuro Peninsula, baits were distributed in a density of 15/km^2^ at an average frequency of 4.3 rounds per year, and prevalence in foxes decreased from 49.4% in 1999 to 15.8% in 2006 (27). However, bait distribution in red foxes is not mandatory in the local municipalities in Hokkaido Prefecture. In 2004, a limited liability company known as the Forum on Environment and Animals was established to conduct *Echinococcus* antigen ELISA and egg examinations for pet animals, in addition to providing advice regarding bait distribution and educational programs on zoonosis (29). The guidance and support of the Hokkaido Institute of Public Health and the Forum on Environment and Animals have resulted in a gradual increase in the number of municipalities participating in bait distribution programs. As of 2017, 13 of 185 municipalities in the prefecture participated in bait distribution (28). The Hokkaido Institute of Public Health also monitors red foxes inhabiting Nemuro Peninsula, an area once heavily infected with *E. multilocularis*. In addition, in 2019, Hokkaido Prefectural Government published a handbook of small-area bait distribution to reduce the risks in parks, universities, and zoos in urban areas, suggesting to distribute a bait each in a 100 m grid square (30).

In order to revitalize echinococcosis control efforts targeting red foxes, local municipalities must be provided with up-to-date scientific knowledge to facilitate evidence-based policy discussions. Potentially useful information for policy discussions includes predictions of red fox populations and fox feces densities. Currently baits are distributed at a same interval on a paved road to achieve a target density in a unit area (27, 30). Various methods have been developed to estimate red fox populations, including determination of the number of breeding dens, the frequency of sightings, footprint density, and feces counting (31, 32). Of these methods, counting feces on paved roads is often employed because it can be done by local municipality officers and serves as a valuable indicator of the risk to humans from exposure to infected feces. However, published methods for feces counting require walking deep inside forested areas (33), which carries the risk of encountering bears. This study was conducted to elucidate the ecological factors associated with the density of red fox feces along paved roads and the relationship between the distributions of red fox populations and fox feces. These data may be useful in designing new echinococcosis control strategies by targeted bait distribution.

## 2 Materials and methods

### 2.1 Study area

The study area was in the central part of Nemuro Peninsula in Hokkaido, Japan, and encompassed approximately 73 km^2^ (Figure 1). The average annual temperature in the study area is 6.6°C, with highest and lowest average temperatures of 17.4°C and − 3.8°C in August and February, respectively. The average annual rainfall over a 30-year period (1991–2020) is 1,040 mm (34). The land is primarily flat, with a highest elevation of 55 m above sea level; the dominant vegetation in the area is pastures, natural grass, and forests (35). Precise geographical locations of fox dens are available only in this area in Hokkaido. Hokkaido Prefecture has the largest number of dairy cattle among 47 prefectures of Japan, which accounted for 59.6% of total dairy cattle population (36). The vegetation is therefore representative of dairy farming areas of the prefecture.

### 2.2 Feces count surveys

Feces count surveys were conducted for a total of 4 years: in 2014, 2016, 2017, and 2018. Each year’s survey consisted of two field projects: cleaning of transects, and counting of feces 2 weeks later to quantify the number of feces accumulated in the transects during the period. Prior to the surveys, all field workers were trained for distinguishing fox feces from other animal feces morphologically and by feeding habit at the Hokkaido Institute of Public Health using photographs and field visits. The photographs of all the feces were taken with identification numbers and geo-coordinates, and doubtful images were checked by the experts. Feces counting was conducted on 1–3 June 2014, 2–3 June 2016, 15–16 May 2017, and 12–13 May 2018, during the season in which red foxes remain with their cubs around the den to ensure a controlled assessment of hazards associated with the most restricted movement pattern of adult foxes during the year. After this season, juveniles, as well as both adult males and females increase range size (37), and it would not be possible to associate feces density data with dens.

Urban foxes, defined as foxes for which part or all of their territory includes urban areas (38), are known to exhibit different behavioral patterns than rural foxes (39–42). In this study, red foxes in suburban and rural areas were targeted, and urban areas were excluded from the surveys.

For setting of the transects, random points were generated using a shapefile, which is a geographical data, of the paved roads, downloaded from the Basic Geospatial Information (43) database using ArcGIS version 10.6.1 (ESRI Japan, Tokyo). Only paved roads were selected for three reasons: (1) foxes mark linear boundaries such as roads and hedges (33), (2) overlooking of feces is more easily avoided, and (3) the risk of encountering bears is lower. The number of random points to be generated was determined by the research team each year so that each survey could be completed within 2 days.

Just before cleaning of feces, both ends of the transect, each of which was 250 m away from the central point in opposite directions along the paved road, were marked with colored spray on the sides of the road. A total length of 500 m along the road both sides was carefully surveyed, and old animal feces were removed. When a length of 500 m was not possible due to factors such as the road being unpaved or damaged, the transects were excluded from the survey.

Two weeks after the initial survey, the transects were revisited using a hand-held GPS device (GPSMAP62SJ, eTrex10J, eTrex30J, eTrex20x, GARMIN), and feces counting was conducted according to the marks previously indicated. The survey range for fox feces was up to 1 m from the edge of and on the pavement. All field investigators were trained in advance to identify fox feces by an expert from the Hokkaido Institute of Public Health. The locations of fox feces were recorded using the hand-held GPS device, and total number of feces in each transect was recorded. The fox feces and the surrounding environment of the transect were also photographed. All recorded feces were regarded as those of adults, as cubs should have stayed around the den. The total number of transects set in 2014, 2016, 2017, and 2018 was 22, 25, 44, and 30, respectively.

### 2.3 Collections of spatial data

**Figure 3:**
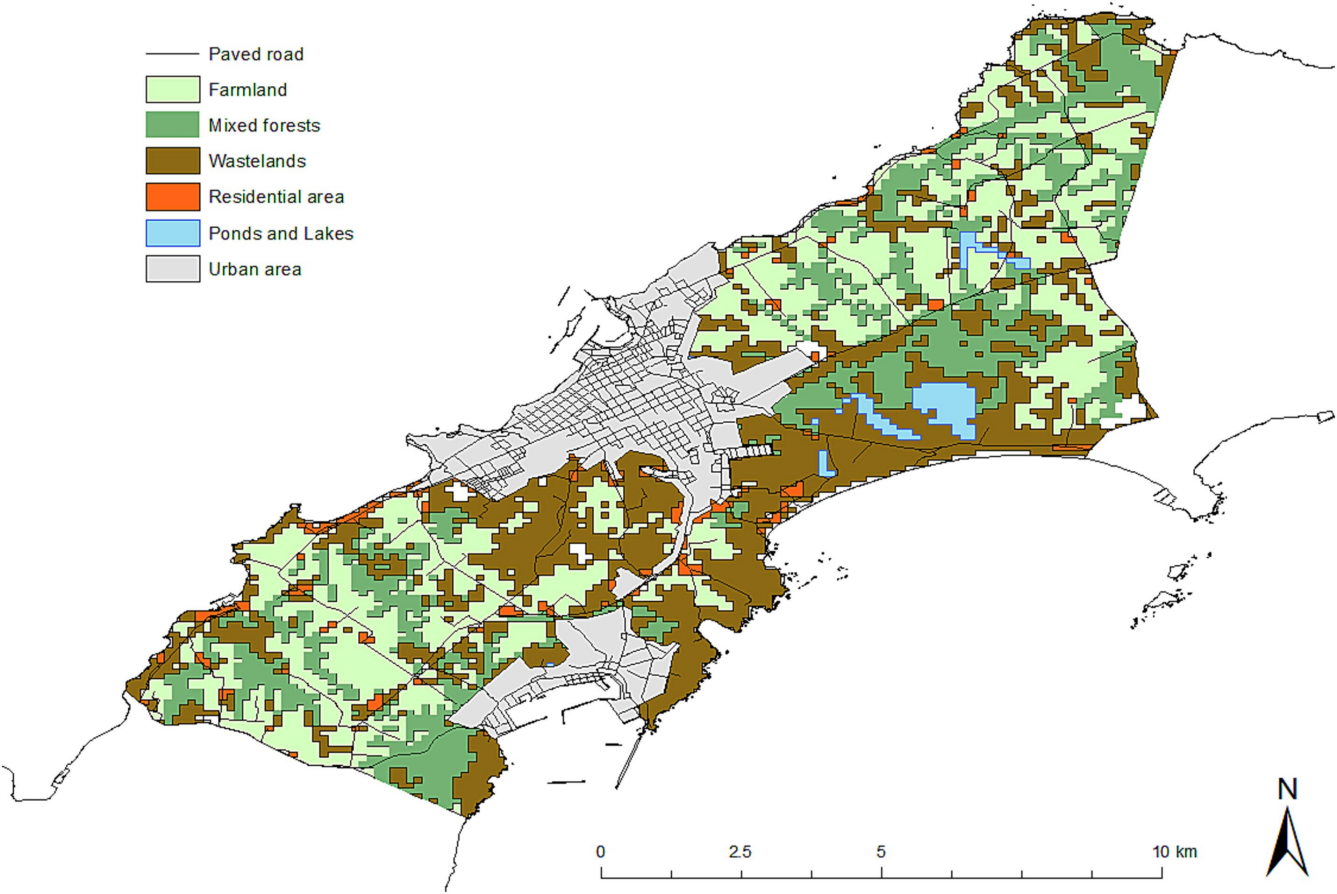
Map of land use categories in the study area.

The geo-coordinates of fox dens in the study years were obtained from the annual monitoring data for Nemuro Peninsula by the Hokkaido Institute of Public Health. The land use data used in this study were downloaded from the National Land Numerical Information website (44). The land use categories for the 100-m grid squares in the study areas included farm land, mixed forest, wasteland, residential areas, and rivers, ponds, and lakes (Figure 3). The degree of slope used for calculations in this study was the value for the 10-m grid square. In the study area, most farm lands are dairy pastures, and based on the vegetation data (45), we refer to dairy farming areas as ‘pasture’ throughout this manuscript. Also, ‘building sites’ are referred to as residential areas in this manuscript to indicate human settlements, as compared to potential territories of foxes. Wastelands included wasteland, cliffs, and rocks.

The Euclidean distances—shortest distances—from a transect to the nearest river, human settlement, livestock farm house (type of animals could not be determined), pasture, and used dens, the distances from used dens to the nearest rivers, and the distances between used dens in the year were calculated using Spatial Analyst in ArcGIS. ArcGIS was also used to calculate the occupancy proportion of each pasture, mixed forest, wasteland, residential area, pond/lake, and steep slope suitable for den sites (35) (slope > 13° within radii of 1 km and 500 m) from the central point of each transect. A slope of >13° was selected based on previous epidemiologic factors evaluated by the authors; the proportion of area > 13° of a circle with 1 km radii was the most positively associated with the number of dens within the circle, using 10 m grid square data for elevation (46) (unpublished). The areas adjacent to each transect were classified ecologically as pasture, mixed forest, wasteland, or residential area (a transect was sometimes adjacent to areas encompassing several different ecological categories). No transect was located by a pond/lake. A residential area for the adjacent ecology of a transect was defined as an area with three or more residential buildings. The adjacent ecological condition of each transect was identified based on satellite images from Google Earth Pro, version 7.3.2.5776 (Google LLC, Mountain View, California) and photographs taken during fieldwork. The number of dens within a 1-km radius from the transect was also calculated using ArcGIS.

### 2.4 Descriptive epidemiology

Spatial distributions of the transects with feces counts and dens used in each year were indicated on a map using ArcGIS. The mean number of feces counted and number of used dens were summarized by year. The shortest distance between used dens was calculated for each year but summarized using 4 years of data.

### 2.5 Ecological factors related to building dens

To characterize the ecological factors related to den building, fox dens were compared with artificially generated control sites. For 131 points where a fox den existed, 262 points were randomly generated using Create Random Points in Geoprocessing in ArcGIS and used as controls. The distance from each point to the river (m), the distance to the road (m), and the slope (degrees) of each point were compared using the Wilcoxon rank-sum test. All shapefiles were downloaded from Basic Geospatial Information (43).

### 2.6 Ecological factor analysis for predicting fox feces counts

Data collected over a 4-year period were used to analyze the ecological factors associated with fox feces counts. Based on Akaike’s information criterion, the error structure, negative binomial error, was selected for fox feces counts from Poisson, negative binomial, zero-inflated Poisson, and zero-inflated negative binomial errors using generalized linear models in the null model.

To generate a variable that was representative of the ecological conditions surrounding the transects, principal component analysis (PCA) was performed to examine the occupancy proportions of the five land use categories within 1 km or 500 m radii from the transects, which were mutually exclusive, using 4 years of accumulated data. The standard deviation, Eigen values, proportion and cumulative proportions of variance of the principal components (PCs) were also calculated. The first PC explains the most variance, and the second PC takes the orthogonal direction to the fist PC which maximizes the remained variance to explain, and so forth. The 1-km and 500-m buffers were selected based on the calculated shortest distances between used dens in a given year (see Results).

Univariable analysis was conducted using continuous domain stochastic partial differential equation (SPDE) in an Integrated Nested Laplace Approximation (INLA) with negative binomial errors. SPDE is a basis-penalty smoother based on the idea that quantities that occur closer together are more similar than those further apart (47), and is applied for point data (48, 49). The residual autoregressive correlation of order 1 (AR1) was selected for the temporal portion to take spatio-temporal autocorrelations into account. AR1 separates the annual variation in fox feces count as random effect from the fixed-effect variables of interest (48, 49). In the analyses, feces count on a transect was selected as the response variable, and ecological factors potentially associated with prey abundance, water availability, den, and human avoidance, such as the principal components for both the 1-km and 500-m buffers, transect ecological category, land use occupancy proportions and slopes >13° degrees within a 1-km radius, and distances from each transect to the nearest river, human settlement, livestock farm house (type of animals was not identifiable), pasture, and used dens were selected as explanatory fixed-effect variables.

Factors with a 95% credible interval (CI) not including zero in the univariable analyses were selected for the multivariable analysis. Collinearity between continuous variables was checked using the variance inflated factor (VIF) to avoid reduced reliability of the model, and there was no pair with a VIF >2. Multivariable SPDE-INLA AR1 models with negative binomial errors were prepared for all combinations of explanatory fixed-effect variables, selecting the feces count on a transect as the response variable. Continuous fixed-effect variables were standardized by dividing their standard deviations. However, inclusion of the PCs from both the 1-km and 500-m buffers in a single model was avoided. The multivariable models were compared based on widely applicable information criteria (WAIC). The R package “INLA” (50) in the statistical software R, version 4.3.0 (51), was used for the analyses.

## 3 Results

### 3.1 Descriptive epidemiology

Table 1 shows the numbers of transects and feces studied, the average number of feces per transect, and used dens over the 4-year study period. The mean and median shortest distances between used dens were 1,347 and 1,368 m, respectively (range: 64–2,919 m; inter-quartile range: 975–1,663 m).

**Table 1:** Numbers of transects, feces, and used dens in 2014–2018.

Year	Transects	Feces count	Mean number of feces	Number of used dens
2014	22	63	2.86	14
2016	25	74	2.96	11
2017	44	143	3.25	13
2018	30	96	3.20	11
Total	121	376	3.11	39^*^

* Overlapped dens used in preceded years were excluded.

**Figure 4:**
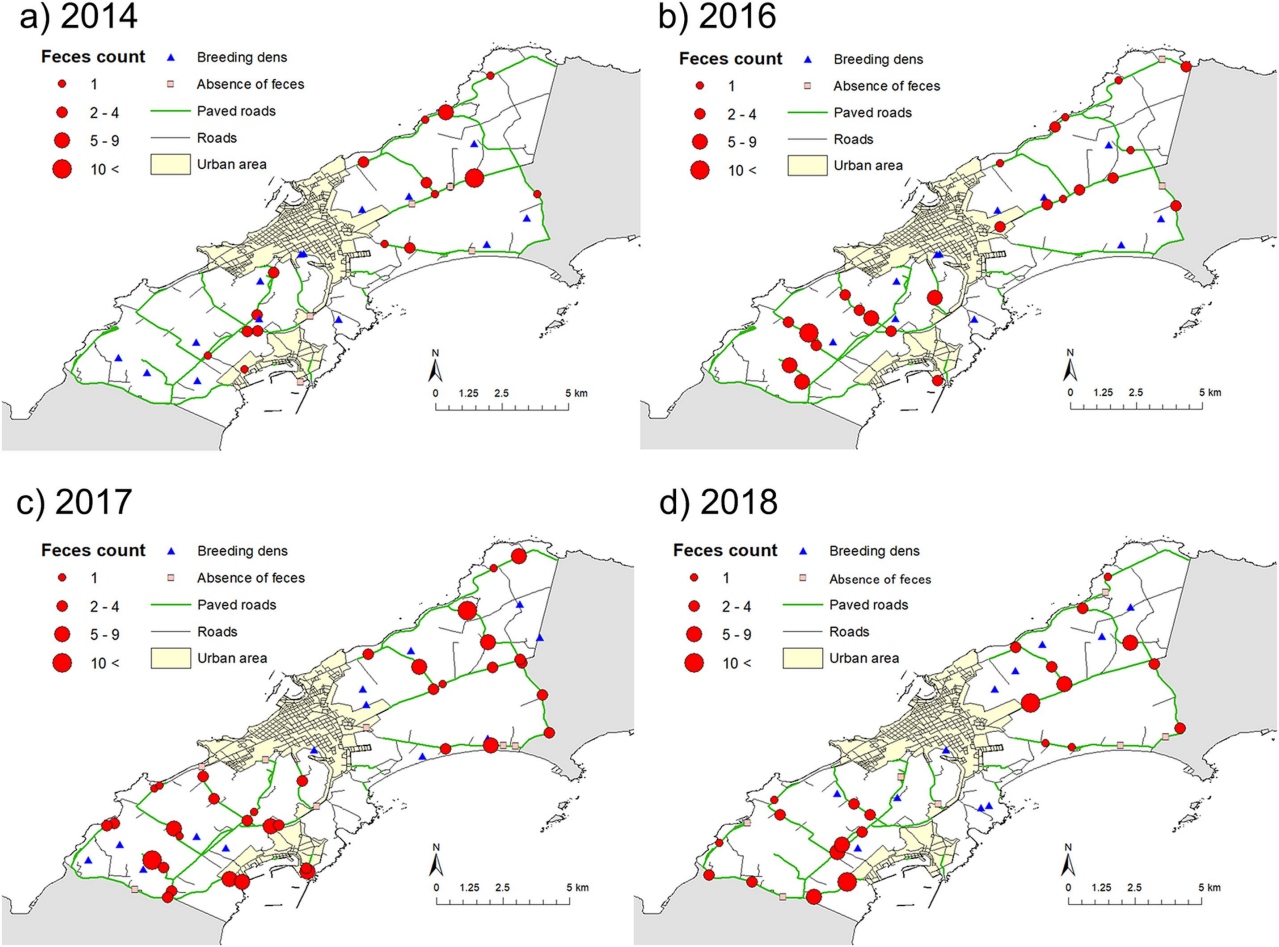
Maps showing the distributions of fox feces counts and used dens. Maps (a–d) show the distributions in 2014, 2016, 2017, and 2018, respectively.

Figure 4 shows the spatial distributions of fox feces, with the number of feces in a transect indicated by the size of the circle. Variation between years was observed, but the higher number of feces in a transect was observed in mixed forests and farm land (pasture) (Figures 3, 4).

### 3.2 Ecological factors related to den building

Table 2 shows a Wilcoxon rank-sum test comparison of the geographic features of dens and controls. The results suggested that red foxes prefer to build dens close to a river (*p* = 0.019), far from roads (*p* = 0.019), and locations with steep terrain (*p* < 0.010).

**Table 2:** Wilcoxon rank-sum test comparison of geographic features of dens and controls.

	Mean		*p-*value
	Dens (*n* = 131)	Control (*n* = 262)	
Distance from nearest river (m)	228	282	0.019
Distance from nearest roads (m)	258	204	<0.010
Slope (degree)	6.76	5.61	<0.010

### 3.3 Ecological factor analysis for predicting fox feces counts

Tables 3, 4 show the results of PCA of the 1-km and 500-m transect buffers, respectively. For the 1-km buffer, the cumulative variance exceeded 70% at PC2, whereas for the 500-m buffer, the cumulative variance exceeded 70% at PC3. Similarly, the Eigen value exceeded 1 in PCs 1 and 2 for the 1-km buffer, and in PCs 1 to 3 for the 500-m buffer. The loading with greater absolute values in both signs (positive or negative) explains the PC more. PC1 of the 1-km buffer was characterized by negative values of loading for pastures and mixed forests. Negative values in PC2 of the 1-km buffer suggested human activities, dairy farming for pastures and human settlements, whereas positive values were associated with natural resources.

**Table 3:** Results of principal component analysis of 1-km buffers.

Land use types	PC1	PC2	PC3
Pasture	−0.52	−0.13	−0.58
Mixed forests	−0.42	0.19	0.78
Wastelands	0.58	0.12	0.01
Human settlements	0.42	−0.56	0.14
Ponds and lakes	0.23	0.79	−0.18
Standard deviation	1.61	1.09	0.91
Eigen value	2.59	1.19	0.84
Proportion of variance	52.1%	23.7%	16.7%
Cumulative proportion	52.1%	75.7%	92.4%

**Table 4:** Results of principal component analysis of 500-m buffers.

Land use types	PC1	PC2	PC3
Pasture	−0.56	−0.26	0.42
Mixed forests	−0.29	0.30	−0.82
Wastelands	0.64	−0.05	0.04
Human settlements	0.25	0.72	0.29
Ponds and lakes	0.37	−0.57	−0.27
Standard deviation	1.50	1.09	1.03
Eigen value	2.25	1.19	1.06
Proportion of variance	44.8%	23.6%	21.1%
Cumulative proportion	44.8%	68.4%	89.5%

The pattern of PC1 of the 500-m buffer was similar to that of the 1-km buffer (i.e., negative values for pastures and mixed forests). PC2 of the 500-m buffer exhibited a strong positive load for human settlement (0.72). PC3 of the 500-m buffer was characterized by a low load of mixed forests (−0.82).

Table 5 shows the results of univariable analysis of the factors associated with fox feces counts. The PC1 values for both the 1-km and 500-m buffers (mean estimates = −0.29 and − 0.33, respectively), which were associated with negative loads for pastures and mixed forests in the PCAs, exhibited negative associations with fox feces count (95% CIs did not include zero). PC3 of the 500-m buffer (mean estimate = −0.24), which was associated with negative loads for mixed forests, was also negatively associated with fox feces count. This suggested that, similarly to PC1, mixed forest occupancy is positively associated with fox feces count.

**Table 5:** Results of univariable analysis of factors associated with fox feces count.

Variables	Mean	95%CI	WAIC
PC1 (1 km buffer)	−0.29	−0.47, −0.11^*^	592.2
PC2 (1 km buffer)	0.12	−0.10, 0.37	600.2
PC1 (500 m buffer)	−0.33	−0.52, −0.15^*^	590.1
PC2 (500 m buffer)	−0.05	−0.27, 0.16	601.0
PC3 (500 m buffer)	−0.24	−0.42, −0.06^*^	593.3
Distance from nearest river	−0.05	−0.21, 0.13	600.7
Distance from nearest used den	−0.01	−0.19, 0.17	601.2
Distance from nearest human settlements	−0.01	−0.19, 0.19	601.6
Distance from nearest livestock farm house	−0.33	−0.51, −0.14^*^	590.7
Distance from nearest pasture	−0.05	−0.22, 0.12	600.7
Transect along pasture	0.38	0.02, 0.75^*^	597.8
Transect along mixed forest	0.55	0.20, 0.91^*^	593.1
Transect along wasteland	−0.46	−0.94, −0.01^*^	597.5
Transect along human settlements	−0.43	−0.78, −0.08^*^	596.4
Proportion of steep area > 13 degree	0.11	−0.05, 0.28	599.6

* 95% credible interval does not include zero.

Significant positive relationships were observed between fox feces count and the categories of transects, pastures, and mixed forests (mean estimates = 0.38 and 0.55, respectively), and feces count was negatively associated with distance to the nearest livestock farm house (−0.33), transects along wastelands (−0.46), and human settlements (−0.43).

Table 6 shows the best five models with the lowest WAIC. No multivariable model including PC1 of the 1-km buffer remained among the 10 models with the lowest WAIC. All five of the best models included PC3 of the 500-m buffer and distance from the nearest livestock farm house. Models 2, 3, and 5 included adjacent ecological categories of the transects; however, none of these ecological categories was significant (95% CIs included zero).

**Table 6:** Multivariable models exhibiting the lowest WAIC.

Model	PC1 (500 m)	PC3 (500 m)	Distance from farm house	Transects along pasture	Transects along mixed forests	Transects along human settlements	Transects along wastelands	WAIC
1		−0.27 (−0.44, −0.10)	−0.35 (−0.53, −0.17)					582.26
2		−0.26 (−0.43, −0.08)	−0.33 (−0.52, −0.14)				−0.29 (−0.73, 0.16)	583.01
3		−0.23 (−0.41, −0.05)	−0.34 (−0.52, −0.15)			−0.24 (−0.59, 0.11)		583.20
4	−0.13 (−0.41, 0.13)	−0.26 (−0.44, −0.08)	−0.25 (−0.52, 0.03)					583.55
5		−0.23 (−0.42, −0.04)	−0.32 (−0.52, −0.12)		0.17 (−0.22, 0.57)			584.07

The best model with the lowest WAIC showed that the density of fox feces was significantly negatively associated with PC3 of the 500-m buffer and distance from the nearest farm house (Tables 6, 7). The random-effects model showed a strong spatial autocorrelation (low standard deviation) and weak temporal autocorrelation (Table 7).

**Table 7:** Final results of the multivariable analysis.

Variables	Mean	2.5th	97.5th
Intercept	1.19	−1.30	3.44
Fixed effects			
PC3 (500 m)	−0.27	−0.44	−0.10
Distance from the nearest farm house	−0.35	−0.53	−0.17
Random effects			
Standard deviation for spatial effect	0.08	0.01	0.42
Rho of temporal effect	0.25	−0.98	0.99

## 4 Discussion

This study was conducted to characterize the ecological factors associated with the density of fox feces along paved roads and the relationship between the distributions of red fox populations and fox feces. The final multivariable model indicated that the density of fox feces could be explained by the surrounding environment within a 500-m radius (i.e., PC3 of 500-m buffers), which was associated with food availability and avoidance of humans. PCA results showed that PC3 of the 500-m buffers was associated with strong negative loading of mixed forests and weak positive loading of human settlements. The negative association between fox feces density and PC3 in the final model suggested that fox feces density is high in areas with high occupancy of mixed forests and few human settlements. The data suggested that red foxes use mixed forests as places to search for food because these areas are inhabited by voles (*Myodes rufocanus bedfordiae*), the main prey of red foxes (35). Red foxes reportedly defecate more in areas in which they spend more time hunting for prey, and they also use feces for scent-marking. A previous study in Spain reported that the density of feces is highest at sites with higher rabbit (prey) population densities, but the locations of feces are associated with landmarks (52). A report on the daily activity patterns in red foxes in Spain found that fox activity is positively associated with prey abundance, and negatively associated with the distance from human settlements, particularly in twilight (after sunrise and before sunset). These relationships are weaker at night (53). Although red foxes are not active during daytime, they exhibit even greater reductions in activity in daytime in areas in which intense fox control measures are implemented (54). Hokkaido Prefecture conducts echinococcosis vector animal epidemiologic surveys (14) particularly in Nemuro Peninsula, and citizens are generally aware of the risk of echinococcosis. Red foxes in the study area may fear human activities, which is commonly seen in many mammalian species (54, 55). Although the transect along human settlements did not remain in the best model in the present study, red fox avoidance of humans could be explained in other alternative models with low WAIC, and the type of environment of the transect is likely associated with feces density. Both prey search and avoidance of humans should be directly associated with survival of red foxes. The animal species of prey for red foxes are different according to the geographical and ecological settings (4); however, dependence of red foxes in their maintenance on prey and human avoidance may be universal.

The PC of the 500-m buffer was an important predictor of feces density, as it was a significant factor in the final multivariable model, whereas the PC of the 1-km buffer was not. This discrepancy can be explained by the mean distance between the nearest used dens, 1,347 m. This suggested that the radius of the home range would be approximately half that distance, or 674 m, slightly above 500 m. Red foxes are territorial animals, with a home range varying from a few hectares to as much as 20–30 km^2^, and overlap of home ranges between fox families is more common among fox species with large ranges than those with small ranges (56). According to a study conducted in the Ashio Mountains in central Japan, the home range of red foxes in the denning period is 108.7 ha (37). Assuming that the home range is circular, the calculated radius in Ashio Mountains would be 588.4 m, which is again comparable to 500 m. This suggests that a home range may be similar in different ecological settings, as parent foxes need to feed cubs in dens. However, caution should be exercised in extrapolations using a 500-m buffer in predicting feces density in the denning period in other areas, as a home range may be influenced by fox population density even in the period.

The density of fox feces was also significantly higher in areas in which the distance to the nearest farm house was shorter. Foxes commonly enter cattle sheds to forage on potential various food sources, including cattle placenta, and the bait application around livestock farms with a caution to avoid accidental ingestion of bait by farm animals has been recommended by the Hokkaido Prefectural Government (57). Other studies have also reported the behavior of red foxes foraging cattle placenta or post-calving discharges, which was associated with the infection with *Mycobacterium bovis* in France (58) and *Neospora caninum* in the United States (59). Cattle placenta can be a favorable prey as less effort is needed to capture than live preys in any country settings.

In the present study, fox dens were characterized as being located close to rivers, far from roads, and on steep terrain. In a previous field study conducted in the same area between June 1986 and May 1987, fox dens were characterized as being located on slopes in woodlands near open spaces and streams but far from human settlements (35). These results suggest that the pattern of den making has not changed in this area.

According to INLA univariable analyses, the distance from the nearest den and the proportion of grid squares with a slope > 13° were not associated with fox feces density. From beginning to mid-May, when the field surveys were conducted, feces were concentrated around den sites, because the fox cubs, which on average are born approximately four cubs per belly, defecate there (60). Transects that were very close to fox dens might have a high density of feces, including those of adult foxes, due to the high amount of time spent near the den to care for the cubs. However, because dens are located far from roads, as demonstrated in this study, counting feces along paved roads may not be sufficiently sensitive to indicate the location of dens. Meso-predators are reported to often travel along roads in winter to conserve energy (61), and red fox in Nemuro Peninsula may also use roads for traveling. There is a report from Spain that the number of red fox feces deposited in clearings was significantly higher than on roads (52), suggesting that the intensity of road use by red foxes is not that high.

Identifying all used dens in the breeding season in a target area would allow estimation of the red fox population, which would be of interest to local public health authorities. Although foxes are more active in areas they densely populate (53), defecation depends on prey seeking, marking (52), and avoidance of disturbances associated with human activities (62). Therefore, precisely estimating red fox populations by counting feces along paved roads may not be achievable. Recently, spatial capture-recapture modeling of non-invasive genetic sampling (NGS) data has been applied to estimate the red fox density in Norway (63). NGS can identify individuals and even sex of red foxes, and that is why biological samples collected in the field can estimate the range sizes of them. Recent molecular researches characterize genetic diversity of *E. multilocularis* and other helminths (64, 65). Integrated spatial analysis of genetic information of foxes and *E. multilocularis* may increase understanding of fox population and infection dynamics of *E. multilocularis*.

The present study provided information useful for reducing the risk of echinococcosis in the study area. Praziquantel bait distribution may be targeted at livestock farms and mixed forests. As the incidence of alveolar echinococcosis is high among dairy farmers and agriculturalists (6), considering the potentially significant land coverage of mixed forests, targeting bait distribution to livestock farms may be an effective public health strategy with high economic efficacy. As red foxes are highly mobile, such targeted bait distribution may result in a reduction in the risk of echinococcosis in peri-urban dwellers as well. The Nemuro City Council follows the guideline for deworming foxes published by Hokkaido Prefecture (57). In practice, baits are distributed along roads at the intervals of either 100 m, 150 m, or 200 m, so that the density of bait exceeds 15 baits per km^2^, avoiding the following areas: (1) where livestock may feed, such as pastures, (2) where agricultural products may be affected, and (3) near natural water sources such as springs, ponds, rivers, and lakes. The current guideline considers even distribution of baits in a target area. To apply the study findings, baits may be distributed at shorter intervals in mixed forest areas, longer in human settlements, and at livestock farms, targeting the areas where red foxes seek prey. Of course, care should be taken in the bait distribution at livestock farms. Moreover, fox dens can be targeted, by choosing the areas with steepness.

This study has three limitations. First, the spatial model used for predicting fox feces counts may not be suitable for other areas in Hokkaido Prefecture or other countries. Red fox is a species highly adapted to diverse ecological conditions (53), and predictive ecological factors may differ in different settings. However, the approach of targeting fox feces can be applied for echinococcosis hazard control considerations in any fox habitat. The most likely difference that may be observed in the statistical results is the PCs significantly associated with fox feces count. In a different climatic or ecological condition, foxes may depend on different animal species or other available foods for prey. Then the loadings of the significant PCs may exhibit ecological characteristics suitable for such prey. Therefore, when this approach is used in the other country or region, it is important to plan how the data representing prey abundance and human avoidance which are suitable for the ecological condition are collected. The second limitation is that this study did not consider urban fox populations. Increased invasion of foxes into urban areas has been reported in several countries (66, 67) and can increase the risk of infection with *Echinococcus* among domestic dogs and, of course, pet owners (68). Food sources associated with households, gardens, and public areas can feed a high number of urban foxes (69). Hegglin et al. (70) reported the successful reduction of *E. multilocularis* coproantigen prevalence in fox feces by targeting bait distribution to sites in which foxes had been seen. As urban areas were excluded in this analysis, the factor associated with fox feces count, mixed forest, would not be a suitable predictor in such areas. Future studies should examine urban risks in greater detail. Spatial analysis can be applied to understand the favorable conditions to make fox dens, prey seeking such as garbage dumping area, and feces count in higher precision. Such information will be helpful in designing small public area bait distribution (23), monitoring *E. multilocularis* prevalence in feces, and evaluation of the disease control. The third limitation is that the level of hazard from infected feces of other mammals such as raccoon dogs is not studied. Raccoon dogs are known to make a pile of feces (71), and pairs can use the same latrines. The method of counting feces on paved roads developed in this study is not applicable to raccoon dogs. Future study for raccoon dogs is needed with new study design.

Although the distribution of praziquantel bait reduces the prevalence of *E*. *multilocularis–*contaminated fox feces, it has the potential to recover, as a trial of over 1.5 years in an urban area showed failure of complete interruption of the life cycle of *E*. *multilocularis* (70). Control strategies for maintaining a low prevalence of *E*. *multilocularis–*contaminated fox feces are thus important. The present study provides a risk-based approach for the control of *E*. *multilocularis*, which should be practically applied under One Health collaboration (25, 26) between different stakeholders.

## Data Availability

The raw data supporting the conclusions of this article will be made available by the authors, without undue reservation.
